# Supplementary material for: The Crystal Structure of Tyrosinase from Verrucomicrobium spinosum Reveals It to Be an Atypical Bacterial Tyrosinase
Source: Biomolecules. 2023 Sep 7;13(9):1360. doi: 10.3390/biom13091360 (PMC10526336; doi:10.3390/biom13091360)
Supplement: Supplementary file 1 [file biomolecules-13-01360-s001.zip › biomolecules-2513845-supplementary.pdf]

Supplementary Information

The crystal structure of tyrosinase from *Verrucomicrobium spinosum* reveals it to be an atypical bacterial tyrosinase

Mostafa Fekry,[1,2] Khyati K. Dave,[1] Dilip Badgujar,[1] Emil Hamnevik,[1] Oskar Aurelius,[3] Doreen Dobritzsch,[1] U. Helena Danielson[1,4] *

[1]Department of Chemistry – BMC, Uppsala University, Uppsala, Sweden
[2]Biophysics Department, Faculty of Science, Cairo University, Giza, Egypt
[3]MAX IV Laboratory, Lund University, Lund, Sweden
[4]Science for Life Laboratory, Drug Discovery & Development Platform, Uppsala University, Uppsala, Sweden
*Communicating author

A)

```
>WP_009958178.1 tyrosinase family protein [Verrucomicrobium spinosum]

1   MSPPTTSRRQ FLVTAGAAAA SAGWSFGQEP AQAATAKYHR LNLQNPAAAP FLESYKKAIT
61  VMLQLPPSDA RNWYRNAFIH TLDCPHGNWW FVVWHRGYTG WFERTVRELS GDPNFAFPYW
121 DWTALPQVPD SFFNGVLDPN NPAFIASYNE FYSQLSNPMS ALWNSFSTAQ LQQMRNRGFQ
181 SVNDVWQAVR DSPMFFPRGR ARTLTRQNPG FDATTRRAVS IGTIRNALAP TDFITFGSGK
241 TANHSESATQ GILESQPHNN VHNNIGGFMQ DLLSPTDPVF FAHHSNIDRL WDVWTRKQQR
301 LGLPTLPTGA NLPLWANEPF LFFIGPDGKP VAKNKAGDYA TIGDFDYNYE PGSGEAVIPA
361 ASRPGEMNNK VWLGTLGAAV PNFSASARAD VMVPEAVPEA AMKADGPAVF AKITIAPPMD
421 VAGVEFHVLV NPPENVSHVD FDSPSFAGTF SVFGKQLGGH KNQPLSFLMP LTEAVKKLQE
481 TNELKPGQPL RVQVVAERKG VNLTPLQAKV SEISVGTF
```

B)

MKSSHHHHHHENLYFQS.

Figure S1. A) Amino-acid sequence of *Verrucomicrobium spinosum* tyrosinase according to NCBI reference sequence WP_009958178.1. The full-length pro-*vs*Tyr is emphasized (red font) and the core domain *vs*Tyr highlighted (yellow). B) Sequence of N-terminal purification tag for the construct used. The non-native serine residue (S) remains at the N-terminus of the protein after the proteolytic cleavage with TEV protease.

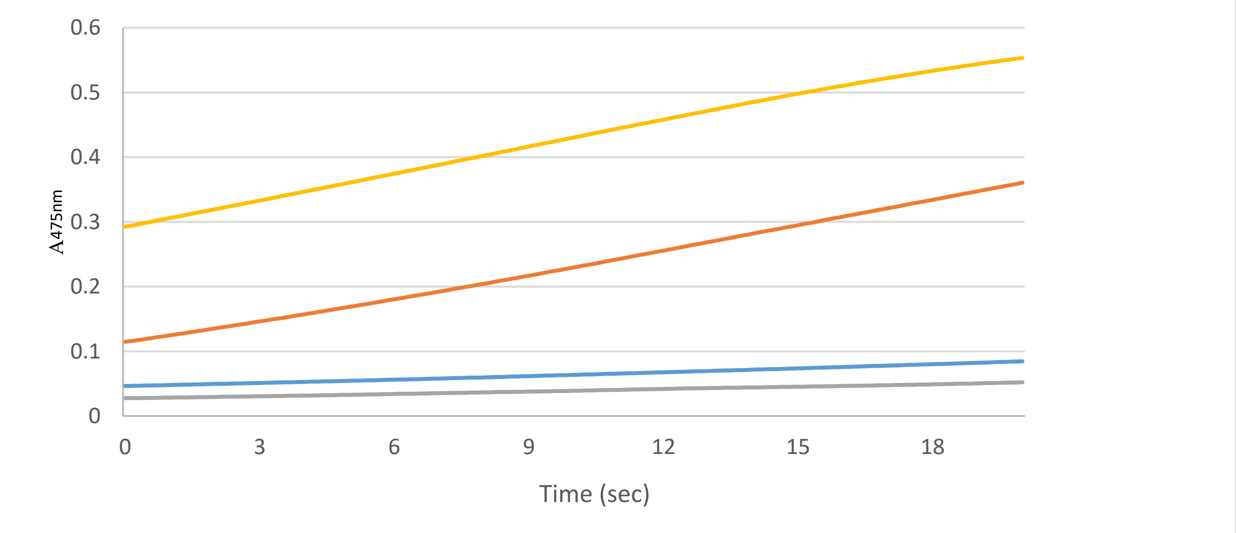

Figure S2. Comparison of the catalytic activity of trypsinated pro-*vs*Tyr and core domain *vs*Tyr with L-DOPA or L-tyrosine as substrate. Trypsinated pro-*vs*Tyr with L-DOPA (blue) and L-tyrosine (orange). Core domain *vs*Tyr with L-DOPA (grey) or L-tyrosine (yellow) as substrate. The rate of dopachrome formation was measured by absorbance at 475 nm in 25 mM potassium phosphate buffer (pH 6.8) with 1 mM of either L-DOPA or L-tyrosine.

Table S1. Comparison of activity of trypsinated pro-vsTyr and core domain vsTyr measuring DOPAchrome formation using 1 mM L-DOPA and L-tyrosine as substrates. Average values from triplicate measurements are given with standard deviations

Enzyme	L-DOPA		L-Tyrosine	
	Activity (µmol/min·mL)	Specific activity (µmol/min·mg)	Activity (µmol/min·mL)	Specific activity (µmol/min·mg)
Trypsinated pro-*vs*Tyr	9.2 ± 0.38<br>8.9[1]	30 ± 1.2<br>29[1]	32 ± 0.33	102 ± 0.89
Core domain *vs*Tyr	17 ± 0.47<br>15[1]	213 ± 5.87<br>184[1]	79 ± 0.92	990 ± 9.8

n.d = not detected

[1] 3 weeks after purification, enzyme stored at 4°C

Table S2. Statistics for anomalous data (used for copper ion occupancy refinement)

**Data Collection**	**Unsoaked crystals**	**$CuSO_4$-soaked crystals**
Space group	C2	C2
a, *b*, *c* (Å), β (°)	84.8 63.4 116.0 96.8	86.3 63.4 117.3 97.2
Molecules in a. u.	2	2
Wavelength (Å)	0.97626	0.97625
Resolution (Å)[a]	115.20-1.43 (1.43-1.56)	116.37-1.64 (1.64-1.67)
Total reflections	426619 (19225)	508362 (15210)
Unique reflections	62664 (3134)	75699 (3073)
Anomalous Multiplicity	3.5 (3.2)	3.4 (2.6)
Anomalous Completeness (%)	93.1 (99.7) (ellipsoidal)<br>55.2 (11.6) (spherical)	97.8 (75.5)
R_{merge}[b] (within I+/I-)	0.096 (0.980)	0.104 (1.876)
R_{meas} (within I+/I-)	0.113 (1.176)	0.124 (2.330)
R_{pim} (within I+/I-)	0.060 (0.641)	0.066 (1.357)
CC(ano)	-0.007 (-0.029)	0.019 (-0.020)
|DANO|/sd(DANO)	0.783 (0.734)	0.780 (0.718)

[a] Values in parentheses are for the highest-resolution shell

Table S3. Copper site occupancy in the *vs*Tyr crystal structures obtained from unsoaked and pre-soaked crystals. Soaking was performed for 15 min in $CuSO_4$ containing reservoir solution prior to data collection. The B-factors of copper ions and average B-factors of the ion-coordinating His residues are also given.

**Copper ID**	**Estimated Cu^{2+} ion occupancy (%) / B-factor (Å^2)**		**Average B-factors (Å^2) of Cu-coordinating His residues**	
	w/o soaking	15 min soak	w/o soaking	15 min soak
CuA (chain A)	43 / 24.4	70 / 42.1	19.1	30.9
CuB (chain A)	78 / 15.6	92 / 25.9	12.8	21.4
CuA (chain B)	40 / 26.5	85 / 47.9	20.2	29.4
CuB (chain B)	80 / 16.4	94 / 30.8	13.2	22.7
Additional sites	-	79 / 59.6 (7 sites)	-	28.8

Table S4. Statistics for structural alignment of the modelled *vs*Tyr C-terminal domain with homologous proteins. The 10 closest structural homologs were identified using the DALI server [1].

PDB-Id (chain)	Description	Z-score	r.m.s.d. [Å][a]	n_{ali}/n_{total}[b]	% Seq id[c]	Reference[d]
7xio (B)	Tyrosinase<br>(*Ralstonia*)	13.8	3.0	142/438	20	Not publ.
6els (A)	Tyrosinase<br>(*Malus domestica*), ***md*Tyr**	11.5	3.5	126/459	19	[2]
4z11 (A)	Aurone synthase<br>(*Coreopsis grandiflora*) ***cg*AUS**	11.1	3.7	129/513	16	[3]
6hqi (A)	Polyphenol oxidase 1<br>(*Solanum lycopersicum*), ***sl*Tyr**	10.1	3.9	1297471	13	[4]
5ta1 (A)	Glycoside hydrolase<br>(*Bacteroidis uniformis*)	9.9	3.2	119/627	9	[5]
4aw7 (A)	GH86A β-porphyranase<br>(*Phocaeicola plebeius*)	9.9	3.1	119/565	9	[6]
6yg8 (A)	Bacterial cellulose secretion regulator BCSB<br>(*E. coli*)	9.5	3.8	115/643	11	[7]
7vbo (A)	Alginate binding domain of alginate lysase<br>(*Defluviitalea phaphyphila*)	9.3	2.9	122/182	6	[8]
5eiy (B)	Putative cellulose synthase<br>(*Cereibacter spharoides 2.4.1*)	8.7	3.3	110/658	17	[9]
1pnf (A)	Peptide-N(4)-(N-acetyl-β-D-glucosaminyl) asparagine amidase F<br>*(Elizabethkingia meningoseptica)*	8.2	3.3	114/314	13	[10]

[a] root mean square deviation
[b] no. aligned Ca atoms/total no. Ca
[c] % amino acid sequence identity
[d] References marked with an asterisk are listed in the main article.

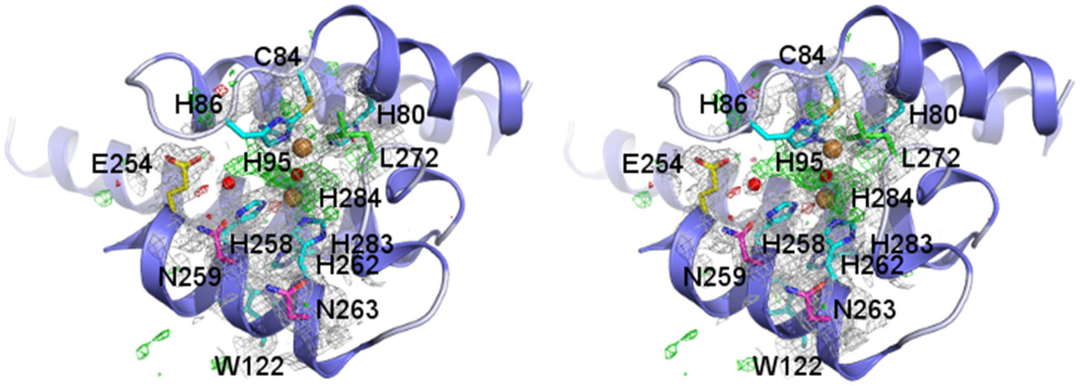

Figure S3. Stereoview of the *vs*Tyr active site. The 4-helix bundle surrounding the metal center is shown as cartoon, copper-coordinating residues, the seventh conserved histidine, H283, and its hydrogen bonding-partner W122 (all with carbon atoms in cyan), the waterkeeper E254 (yellow), the activity controllers N259 and N263 (magenta), and L272 (green) as sticks. The latter corresponds to the position of the gatekeeper residue as seen in plant PPOs (see also figure S3). CuA and CuB are shown as orange spheres, catalytically important water molecules as red spheres. C84 and H86 are covalently linked via a thioether bond. The final 2Fo-Fc map (grey mash) is contoured at 1σ, and the Fo-Fc map at 3σ (green) and -3σ (red), within a radius of 1 Å around all depicted residues.

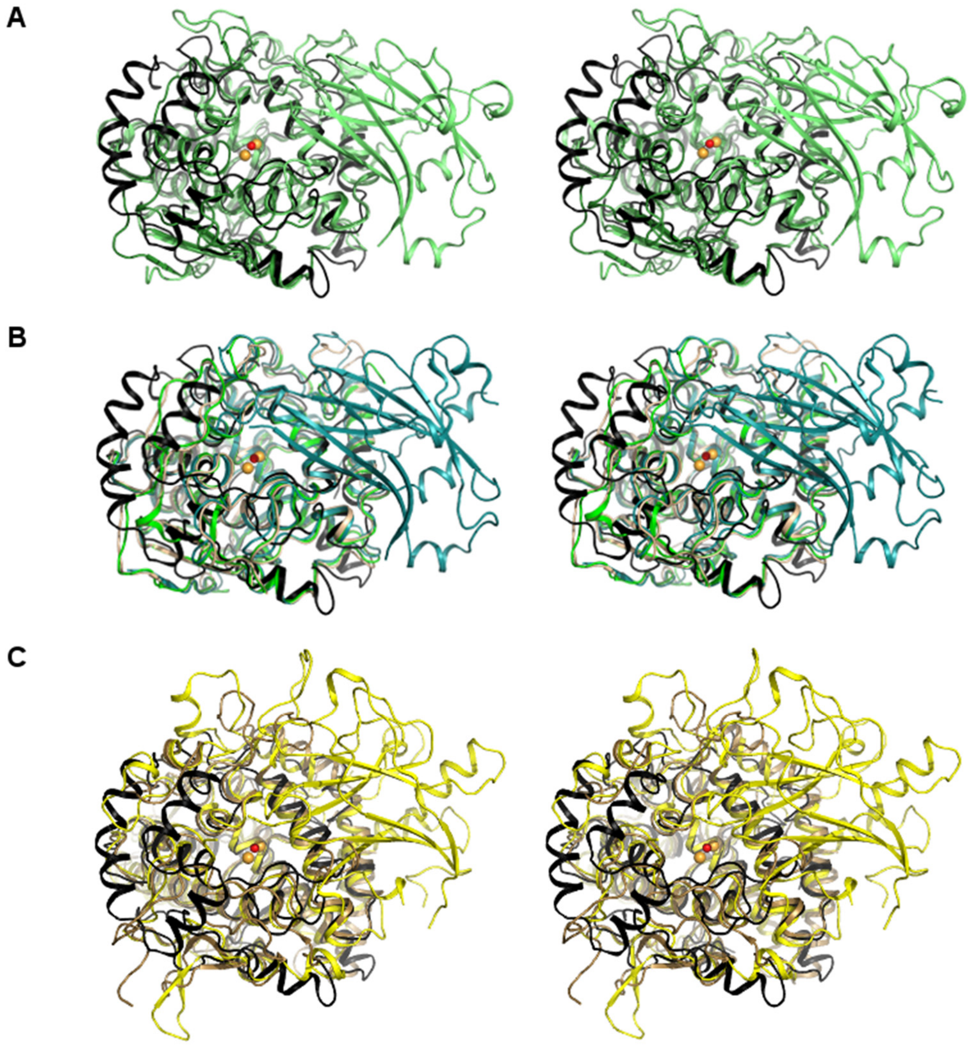

Figure S4. Stereo views of structural superimpositions of *vs*Tyr with other members of the type III copper protein family (see Table 2 for full names, PDB IDs, and references). The *vs*Tyr core domain structure in cartoon representation is colored black, and the copper ions and bridging water molecule are shown as spheres in orange and red, respectively (in all panels). The copper centers of the homologous enzymes are not shown. (**A**) and (**B**) *Vs*Tyr superimposed with plant-derived catechol oxidases and tyrosinases. In (**A**) *sl*Tyr (lime green), *ib*CO (forest green). In (**B**): *vv*Tyr (beige), *jr*Tyr (green), *md*Tyr (deep teal). (**C**) *Vs*Tyr superimposed with fungal tyrosinases: *ab*Tyr (light brown), *ao*Tyr, (yellow).

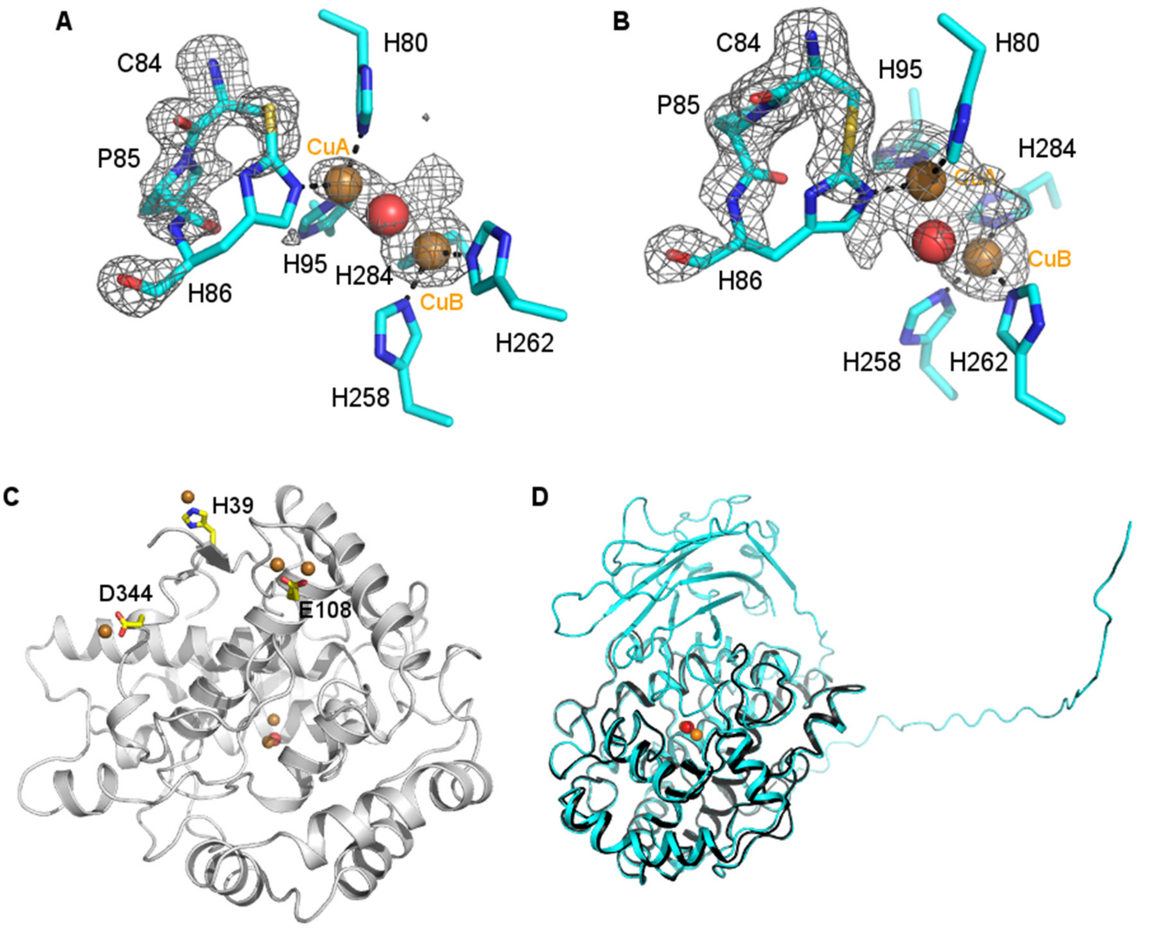

Figure S5. *Vs*Tyr copper binding sites and model of full-length *vs*Tyr. (**A**) and (**B**) The binuclear metal site in chain A of the *vs*Tyr structures obtained with unsoaked (**A**) and $CuSO_4$-soaked crystals (**B**). The copper ions and bridging water molecule are shown as spheres, the metal-coordinating histidine residues, C84 and P85 as sticks. An omit map obtained after exclusion of the metal site and residues 84-86 from the structural model is shown in grey at a contour level of 3σ. (**C**) Location of additional copper ion binding sites (orange spheres) in the *vs*Tyr core domain structure determined using $CuSO_4$-soaked crystals. The unlabeled site corresponds to the binuclear center in the active site. (**D**) Superimposition of the experimentally determined *vs*Tyr core domain structure (black) with the structural model of full-length *vs*Tyr predicted by AlphaFold [11,12].

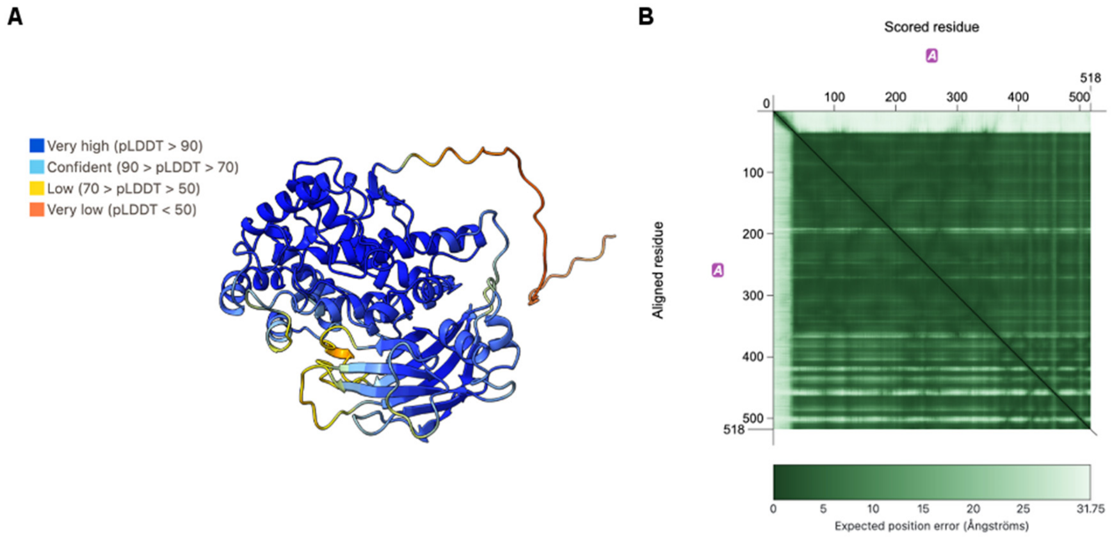

Figure S6. AlphaFold prediction of the structure of full-length *vs*Tyr [11,12]. (**A**) Cartoon of the predicted full-length *vsTyr* colored according to the pLDDT score. (**B**) Plot of the predicted aligned error (PAE).

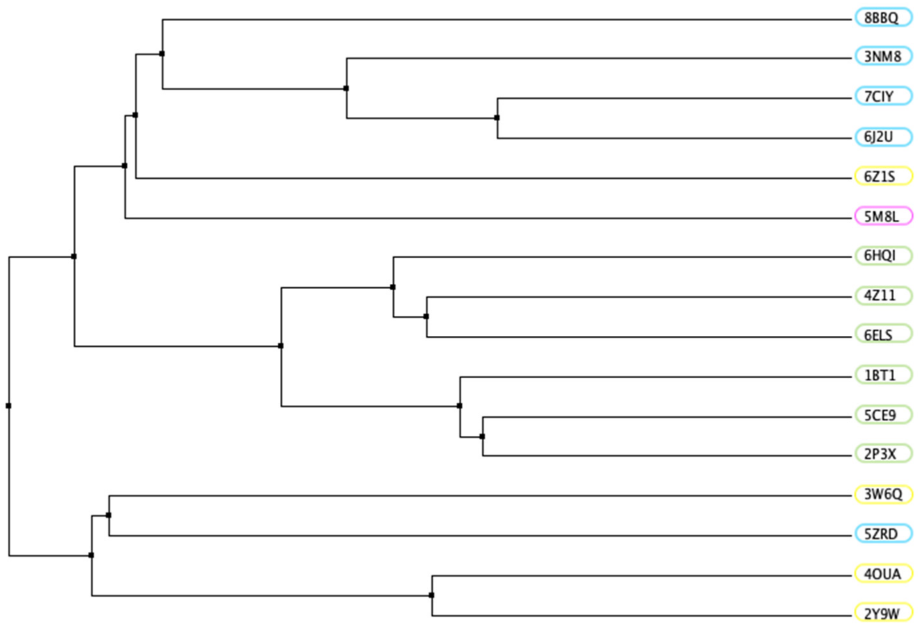

Figure S7. Phylogenetic tree for the proteins mentioned in Table 2 and their evolutionary relation to *vs*Tyr (8BBQ). The phylogenetic tree was generated using average distance method (BLOSUM52) by Jalview software [13]. The structures are surrounded are framed with the same color code used in Table 2 for different phylogenetic groups (Blue: bacterial, green: plant, yellow: fungal, pink: Human)

Figure S8. Pairwise sequence alignment of all protein structures listed in Table 2 with *vs*Tyr core domain (8BBQ). The alignments were produced using EMBL-EBI analysis tools [14].

a) Tyrosinase from *Burkholderia thailandensis*

```
8BBQ               1 ------------------AKYHRLNLQNPAAAPFLESYKKAITVMLQLPP     32
                                       .:|     .|....| ||...:|...:..|||
5ZRD               1 MGSNVNAPRVRRSVRDLQKRY-----DNGEKKP-LEDLVRAWVGIQALPP     44

8BBQ              33 SDARNWYRNAFIH--------TLD--------------CPHGNWWFVVWH     60
                     ||.::::.....|        .:|              |.|||..|..||
5ZRD              45 SDPKSFFALGGYHGEPFQYRKPVDALPQDDIYPYWGGYCNHGNVLFPTWH     94

8BBQ              61 RGYTGWFERTVRELSGDPNFAFPYWDWT-------ALPQV--PDSFFNGV    101
                     |.|....|..::.:.  |..:.|:||.|       .:|.:  .:.|....
5ZRD              95 RMYVYKLEEALQSIV--PGVSMPFWDETDEYTLKHGIPSILTQEKFELDG    142

8BBQ             102 LDPNNP--AFIAS-------------YN--EFYSQLSNPMSALWNSFSTA    134
                     ...:||  :|:..             |.  :.|..:..|:|.|..:....
5ZRD             143 KQIDNPLRSFVLPVALSDRLPGDGNIYEKPKGYVTVRYPLSGLVGTPEAL    192

8BBQ             135 QLQQMRNRGFQSVNDVWQAVRDSPMFFPRGRARTLTRQNPGFDATTRRAV    184
                     :..::.|..|.......:.:.:....:.:|.:.|     ||....||..|
5ZRD             193 EQTKIHNAKFPLPEKNTELLNSNVRAWLKGDSPT-----PGDPDPTRNGV    237

8BBQ             185 SIGTIRNALAPTDFITFGSGKTANHSESATQGILE--SQPHNNVHNNIGG    232
                     ....:|...|| ::..|.:..:|:...|:..|::.  ..|||::|..:||
5ZRD             238 YAKYVRCLSAP-NYTVFSNTTSASVWNSSNPGLVTPVESPHNDIHLAVGG    286

8BBQ             233 F-----------------MQDLLSPTDPVFFAHHSNIDRLWDVW---TRK    262
                     |                 .::..:..||:||.||.|:||::.||   |..
5ZRD             287 FDYGGDEIGQIAGANGDMGENNTAGMDPIFFFHHCNVDRMFWVWQKQTGH    336

8BBQ             263 QQRLGL----------------PTLPTGANL----PLWANEPFLFFIGPD    292
                     ..||.:                |....|.:|    ||   .||
5ZRD             337 TDRLDIIRNYPGTNASDSQGPTPGFAPGESLNLTTPL---NPF-------    376

8BBQ             293 GKPVAKNKAGDYATIGD-------FDYNYEPGSGEAV-------------    322
                          |..:|:..|..|       ..:.|.|||.:..
5ZRD             377 -----KKASGEAYTSEDCINIERQLGFTYGPGSLDDATPELKSLLAVPSG    421

8BBQ             323 --------------------------------------------------    322

5ZRD             422 NSTKKLTVTGIDRAQIQGSFIMKAYASVTDANGKTREYYLGHKSILSRWN    471

8BBQ             323 --------------------------------------------------    322

5ZRD             472 VVQCANCLTHLDIVAHFPLSAMPADDVPKAKFRVEFIHRGGGVPSAAKAA    521

8BBQ             323 ----------------------------    322

5ZRD             522 IDKVSALQPKFEVSDKLAAALEHHHHHH    549
```

b) Tyrosinase from *Bacillus megaterium*

```
8BBQ                   1 --------------------------------AKYHRLNLQNPAAAPFLE     18
                                                         ..|.|....:.||..|
3NM8                   1 MSNKYRVRKNVLHLTDTEKRDFVRTVLILKEKGIYDRYIAWHGAAGKF--     48

8BBQ                  19 SYKKAITVMLQLPPSDARNWYRNAFIHTLDCPHGNWWFVVWHRGYTGWFE     68
                                   ..||...||           ..|.:..|:.|||.|...||
3NM8                  49 ----------HTPPGSDRN-----------AAHMSSAFLPWHREYLLRFE     77

8BBQ                  69 RTVRELSGDPNFAFPYWDWTALPQVPDSFFNGVLDPNNPAFIASYNEFYS    118
                         |.::.:  :|....|||:|                           |..:
3NM8                  78 RDLQSI--NPEVTLPYWEW---------------------------ETDA     98

8BBQ                 119 QLSNP-MSALWNSFSTAQLQQMRNRGFQSVNDVWQAVRDS-PMFFPRGRA    166
                         |:.:| .|.:|::      ..|...|    |.:....:.|: |  |..||.
3NM8                  99 QMQDPSQSQIWSA------DFMGGNG----NPIKDFIVDTGP--FAAGRW    136

8BBQ                 167 RTLTRQ-NP------GFDATTRRAVSIGT---IRNALAPTDFITFGSGKT    206
                         .|:..| ||      .|.| |:.|.::.|   :.|||..|.:.|.....|
3NM8                 137 TTIDEQGNPSGGLKRNFGA-TKEAPTLPTRDDVLNALKITQYDTPPWDMT    185

8BBQ                 207 ANHS-ESATQGILES-QPHNNVHNNIGGFMQDL-LSPTDPVFFAHHSNID    253
                         :.:| .:..:|.:.. |.||.||..:||.|..: .:|.|||||.||:|:|
3NM8                 186 SQNSFRNQLEGFINGPQLHNRVHRWVGGQMGVVPTAPNDPVFFLHHANVD    235

8BBQ                 254 RLWDVWT---RKQQRLGLPTLPTGANL--PL--WANEPFLFFIGPDGKPV    296
                         |:|.||.   |.|....:...|.|.|.  |:  |...|         :.|
3NM8                 236 RIWAVWQIIHRNQNYQPMKNGPFGQNFRDPMYPWNTTP---------EDV    276

8BBQ                 297 AKNKAGDYATIGDFDYNYEPGSGEAV-------    322
                         ..::...|.      |:.|....:..
3NM8                 277 MNHRKLGYV------YDIELRKSKRSSHHHHHH    303
```

c) Tyrosinase from *Streptomyces avermitilis*

```
8BBQ                    1 --AKYH-----RLNLQNPAAAPFLESYKKAITVMLQLPPSDARNWY---R     40
                            :.:|     |...:|.|... .:..::.:..::.|..|...:.:   .
6J2U                    1 MGSHHHHHHSERTVRKNQATLT-ADEKRRFVDALVALKRSGRYDEFVTTH     49

8BBQ                   41 NAFI--------HTLDCPHGNWWFVVWHRGYTGWFERTVRELSGDPNFAF     82
                          ||||        .|   .|.:..|:.|||.:...||:.::.:  ||:.|.
6J2U                   50 NAFIMGDTDSGERT---GHRSPSFLPWHRRFLIEFEQALQAV--DPSVAL     94

8BBQ                   83 PYWDWT------ALPQVPDSFFNG--------VLDPNNPAFIASYNEFYS    118
                          |||||:      |....|| |..|        |:|
6J2U                   95 PYWDWSTDRTARASLWAPD-FLGGSGRSLDGRVMD--------------    128

8BBQ                  119 QLSNPMSALWNSFSTAQLQQMRNRGFQSVNDVWQAVR-DSPMFFPRGRAR    167
                             .|.:|              :.|...||     || ||..:..|
6J2U                  129 ---GPFAA--------------STGNWPVN-----VRVDSRTYLRR----    152

8BBQ                  168 TLTRQNPGFDATTRRAVSIGTIRNALAPTDFITFGSGKTA--NHSESATQ    215
                            |....|.:..||..|....   |::..|...:.|....  ||.| ..:
6J2U                  153 --TLGGGGRELPTRAEVDSVL---AMSTYDMAPWNSASDGFRNHLE-GWR    196

8BBQ                  216 GILESQPHNNVHNNIGGFMQDLLSPTDPVFFAHHSNIDRLWDVWTRKQQR    265
                          |:   ..||.||..:||.|...:||.||||:.||:.|||||..|..:...
6J2U                  197 GV---NLHNRVHVWVGGQMATGVSPNDPVFWLHHAYIDRLWAQWQSRHPG    243

8BBQ                  266 LGLPTLPTGANLPLWANEPFLFFIGPDGKPVAKNKAGD---------YAT    306
                          .|.  :|||..       |.:..:....||....:..|         :.|
6J2U                  244 SGY--VPTGGT-------PNVVDLNETMKPWNDVRPADLLDHTAHYTFDT    284

8BBQ                  307 IGDFDYNYEPGSGEAV    322
                          :
6J2U                  285 V---------------    285
```

d) Tyrosinase from *Streptomyces castaneoglobisporus*

```
8BBQ                     1 AKYHRLNLQNPAAAPFLESYKKAITVMLQLPPSDARNWY---RNAFIHTL     47
                                :.::...|....:..::.:..:|:|..|...:.:   .|.||.: 
7CIY                     1 -----MTVRKNQATLTADEKRRFVAAVLELKRSGRYDEFVRTHNEFIMS-     44

8BBQ                    48 DCP------HGNWWFVVWHRGYTGWFERTVRELSGDPNFAFPYWDWTALP     91
                           |..      |.:..|:.|||.:...||:.::  |.|.:...|||||:|..
7CIY                    45 DTDSGERTGHRSPSFLPWHRRFLLDFEQALQ--SVDSSVTLPYWDWSADR     92

8BBQ                    92 QVPDSFFNGVLDPNNPAFIASYNEFYSQLSNPMSALWNSFSTAQLQQMRN    141
                           .|..|.:       .|.|:.                    .|.:....| 
7CIY                    93 TVRASLW-------APDFLG--------------------GTGRSTDGR-    114

8BBQ                   142 RGFQSVNDVWQAVRDSPMFFPRGR--------ARTLTRQNPG---FDATT    180
                                       |.|.|.....|.        :||..|::.|   .:..|
7CIY                   115 ------------VMDGPFAASTGNWPINVRVDSRTYLRRSLGGSVAELPT    152

8BBQ                   181 RRAVSIGTIRNALAPTDFITFGSGKTA--NHSESATQGILESQPHNNVHN    228
                           |..|....   |::..|...:.|....  ||.| ..:|:   ..|..||.
7CIY                   153 RAEVESVL---AISAYDLPPYNSASEGFRNHLE-GWRGV---NLHGRVHV    195

8BBQ                   229 NIGGFMQDLLSPTDPVFFAHHSNIDRLWDVWTRKQQRLGLPTLPTGANLP    278
                           .:||.|...:||.||||:.||:.:|:||..|.|:.        |..|.:|
7CIY                   196 WVGGQMATGVSPNDPVFWLHHAYVDKLWAEWQRRH--------PDSAYVP    237

8BBQ                   279 LWANEPFLFFIGPDGKPVAKNKAGD------YATIGDFDYNYEPGSGEAV    322
                           . ...|.:..:....||....:..|      |.|....::::...     
7CIY                   238 T-GGTPDVVDLNETMKPWNTVRPADLLDHTAYYTFDALEHHHHHH-----    281
```

e) Aurone synthase from *Coreopsis grandiflora*

```
8BBQ                 1 ---------------------------------------------AKYHR      5
                                                                    ....:
4Z11                 1 APITAPDITSICKDASSGIGNQEGAIRTRKCCPPSLGKKIKDFQFPNDKK     50

8BBQ                 6 LNLQNPA---AAPFLESYKKAITVMLQLPPSDARNWYRNAFIHTLDCP--     50
                       :.::.||   ....::.|::||..|..||..|.|::...|.||...|.
4Z11                51 VRMRWPAHKGTKKQVDDYRRAIAAMRALPDDDPRSFVSQAKIHCAYCNGG    100

8BBQ                51 ---------------HGNWWFVVWHRGYTGWFERTVRELSGDPNFAFPYW     85
                                      |.:|.|..:||.|..::||.:..|..:||||.|||
4Z11               101 YTQVDSGFPDIDIQIHNSWLFFPFHRWYLYFYERILGSLIDEPNFALPYW    150

8BBQ                86 DWTALPQVPDSFFNGVLDPNNPAFIASYNEFYSQLSNPMSALWNSFSTAQ    135
                       .|.....:|.|                 |.|....|||   |::.:..|
4Z11               151 KWDEPKGMPIS-----------------NIFLGDASNP---LYDQYRDA-    179

8BBQ               136 LQQMRNRGFQSVNDVWQAVRDSPMFFPRGRARTLTRQNPGFDATTRRAVS    185
                                   |.:...:.|...   .|:.:.:..|.       :.|.:
4Z11               180 ------------NHIEDRIVDLDY---DGKDKDIPDQQ-------QVACN    207

8BBQ               186 IGT-----IRNALAPTDFITFGSGKTANHSESA----TQGILESQPHNNV    226
                       :.|     :||.:.||.|  ||....|..|..|    :.|.:|:..|..|
4Z11               208 LSTVYRDLVRNGVDPTSF--FGGKYVAGDSPVANGDPSVGSVEAGSHTAV    255

8BBQ               227 HNNIGGFMQDLLSPT---------------DPVFFAHHSNIDRLWDVWTR    261
                       |..:|       .||               ||||:.||:|:||:|.:|
4Z11               256 HRWVG-------DPTQPNNEDMGNFYSAGYDPVFYIHHANVDRMWKLW--    296

8BBQ               262 KQQRLGLPTLPTGANL--PLWANEPFLFFIGPDGKPVAKNKAGDYATIGD    309
                       |:.|     ||...::  |.|.|..::|:  .:.|.:.:....|...:..
4Z11               297 KELR-----LPGHVDITDPDWLNASYVFY--DENKDLVRVYNKDCVNLDK    339

8BBQ               310 FDYNYEPGSGEAV-------------------------------------    322
                       ..||:...|.|..
4Z11               340 LKYNFIENSKEVFPWRNSRPPQRRKSAQVATTGDVKTVEQTKFPVRLNQI    389

8BBQ               323 --------------------------------------------------    322

4Z11               390 FKVRVKRPAVNRTEEEKDQANEVLLIKKIKYDSGKFVKFDVFVNDKLKDG    439

8BBQ               323 --------------------------------------------------    322

4Z11               440 VFTTPCDPEYAGGFAQIPHNDKRSMVMTSTARFGLNELLEDTNTEGEEYA    489

8BBQ               323 ----------------------------    322

4Z11               490 TVTLVPRTGCEDLTVGEIKIELVPIPKA    517
```

f) Polyphenol oxidase 1 from *Solanum lycopersicum*

```
8BBQ                 1 ------------------------------------------AKYHRLNL      8
                                                                 ....:|.:
6HQI                 1 APIPPPDLSSCNKPKINATTEVPYFCCAPKPDDMSKVPYYKFPSVTKLRI     50

8BBQ                 9 QNPAAA---PFLESYKKAITVMLQL---PPSDARNWYRNAFIHTLDC---     49
                       :.||.|   .::..|..||:.|..|   .|.:...:.:.|.||...|
6HQI                51 RPPAHALDEAYIAKYNLAISRMKDLDKTQPDNPIGFKQQANIHCAYCNGG    100

8BBQ                50 ---------PHGNWWFVVWHRGYTGWFERTVRELSGDPNFAFPYWDWTAL     90
                                .|.:|.|..:||.|..::||.:..|..||.|..|:|:|..
6HQI               101 YSIDGKVLQVHNSWLFFPFHRWYLYFYERILGSLIDDPTFGLPFWNWDH-    149

8BBQ                91 PQVPDSFFNGVLDPNNPAF----IASYNEFY-SQLSN----PMSALWNSF    131
                       |:       |:..|  |.|    .|.|:|.. .|:.|    .:.:..:..
6HQI               150 PK-------GMRFP--PMFDVPGTALYDERRGDQIHNGNFIDLGSFGDQV    190

8BBQ               132 STAQLQQMRNRGFQSVNDVW-QAVRDSP---MFFPRGRARTLTRQNPGFD    177
                       .|.|||.|.|    ::..:: |.|.:||   |||  |...||
6HQI               191 ETTQLQLMTN----NLTLMYRQLVTNSPCPLMFF--GGPYTL--------    226

8BBQ               178 ATTRRAVSIGTIRNALAPTDFITFGSGKTANHSESATQGILESQPHNNVH    227
                                                 |.|.   |:|  |.:|:.||:.||
6HQI               227 --------------------------GSTV---EAA--GTVENIPHSPVH    245

8BBQ               228 -------------------NNIGGFMQDLLSPTDPVFFAHHSNIDRLWDV    258
                                          .::|.|..   :..||:|:.||||:||:|:.
6HQI               246 IWVGTRRGSVLPDGKISNGEDMGNFYS---AGLDPLFYCHHSNVDRMWNE    292

8BBQ               259 WTRKQQRLGLPTLPTGANLPL----WANEPFLFFIGPDGKPVAKNKAGDY    304
                       |    :..|      |....|    |.|..| ||...:|.|. |.:..|.
6HQI               293 W----KATG------GKRTDLQNKDWLNSEF-FFYDENGNPF-KVRVRDC    330

8BBQ               305 ATIGDFDYNYEPGSGEAV--------------------------------    322
                       .......|:|:|   .|.
6HQI               331 LDTKKMGYDYQP---TATPWRNFKPKTKASAGKVNTGSIPPESQVFPLAK    377

8BBQ               323 --------------------------------------------------    322

6HQI               378 LDKAISFSINRPASSRTQQEKNAQEEVLTFNAIKYDNRDYIRFDVFLNVD    427

8BBQ               323 --------------------------------------------------    322

6HQI               428 NNVNANELDKAEFAGSYTSLPHVHRVGDPKHTATATLRLAITELLEDIGL    477

8BBQ               323 -----------------------------    322

6HQI               478 EDEDTIAVTLVPKKGDISIGGVEIKLADC    506
```

g) Catechol oxidase from *Ipomoea batatas*

```
8BBQ                1 --------------------------------------------------      0

1BT1                1 APIQAPEISKCVVPPADLPPGAVVDNCCPPVASNIVDYKLPAVTTMKVRP     50

8BBQ                1 AKYHRLNLQNPAAAPFLESYKKAITVMLQLPPSDARNWYRNAFIHTLDC-     49
                      |.:   .:...|.|.|    .||:.:|..||..|.||:|:.|.:|...|
1BT1               51 AAH---TMDKDAIAKF----AKAVELMKALPADDPRNFYQQALVHCAYCN     93

8BBQ               50 --------------PHGNWWFVVWHRGYTGWFERTVRELSGDPNFAFPYW     85
                                    .|.:|.|..:||.|..::||.::.|.|||:|..|:|
1BT1               94 GGYDQVNFPDQEIQVHNSWLFFPFHRWYLYFYERILGKLIGDPSFGLPFW    143

8BBQ               86 DWTALPQVPDSFFNGVLDPNNPAFIASYNEFYSQLSNPMSALWNSFSTAQ    135
                      :|                 :||..:. ..:|   |::..|:|::|
1BT1              144 NW-----------------DNPGGMV-LPDF---LNDSTSSLYDS-----    167

8BBQ              136 LQQMRNRGFQSVNDVWQAVRDSPMFFPRGRARTLTRQNPGFDATTRRAVS    185
                           ||....:..|   |.|            |.......|.|.::.:
1BT1              168 -----NRNQSHLPPV---VVD------------LGYNGADTDVTDQQRI-    196

8BBQ              186 IGTIRNALAPTDFIT--------FGSGKTANHSESATQGILESQPHNNVH    227
                        |...||.....:|        .|....|..:.|...|.:|:.||..:|
1BT1              197 --TDNLALMYKQMVTNAGTAELFLGKAYRAGDAPSPGAGSIETSPHIPIH    244

8BBQ              228 ---------NN--IGGFMQDLLSPTDPVFFAHHSNIDRLWDVWTRKQQRL    266
                               ||  :|.|..   :..|..|:.||||:||:|.:|   ||..
1BT1              245 RWVGDPRNTNNEDMGNFYS---AGRDIAFYCHHSNVDRMWTIW---QQLA    288

8BBQ              267 GLPTLP--TGANLPLWANEPFLFFIGPDGKPVAKNKAGDYATIGDFDYNY    314
                      |.|...  |.::   |.|..|||: ..:|:.| |.:.||........|.|
1BT1              289 GKPRKRDYTDSD---WLNATFLFY-DENGQAV-KVRIGDSLDNQKMGYKY    333

8BBQ              315 ----------EPGSGEAV    322
                                :|
1BT1              334 AKTPLPWLDSKP------    345
```

h) Tyrosinase from *Vitis vinifera*

```
8BBQ                       1 ------------------------------------------------AK      2
                                                                             ..
2P3X                       1 APIQAPDISKCGTATVPDGVTPTNCCPPVTTKIIDFQLPSSGSPMRTRPA     50

8BBQ                       3 YHRLNLQNPAAAPFLESYKKAITVMLQLPPSDARNWYRNAFIHTLDCP--     50
                             .|.::.:      :|..|||||.:...||..|.|::.:.|.:|...|.
2P3X                      51 AHLVSKE------YLAKYKKAIELQKALPDDDPRSFKQQANVHCTYCQGA     94

8BBQ                      51 -------------HGNWWFVVWHRGYTGWFERTVRELSGDPNFAFPYWDW     87
                                          |.:|.|:.:||.|..:.||.:.:|..||.||.|||.|
2P3X                      95 YDQVGYTDLELQVHASWLFLPFHRYYLYFNERILAKLIDDPTFALPYWAW    144

8BBQ                      88 TALPQVPDSFFNGVLDPNNPAFIASYNEFYSQLSNPMSALWNSFSTAQLQ    137
                                 ..||..:...:..::|:.:  |:|..:.....|.:.:...:...:
2P3X                     145 ----DNPDGMYMPTIYASSPSSL--YDEKRNAKHLPPTVIDLDYDGTE--    186

8BBQ                     138 QMRNRGFQSVNDVWQAVRDSPMFFPRGRARTLTRQNPGFDATTRRAVSIG    187
                                           ..:.|..:                  .|...|:...
2P3X                     187 --------------PTIPDDEL------------------KTDNLAIMYK    204

8BBQ                     188 TIRNALAPTDFITFGSGKTANHSESATQGILESQPHNNVH----------    227
                             .|.:. |.|..:..|....|..:.....|.||..|||.||
2P3X                     205 QIVSG-ATTPKLFLGYPYRAGDAIDPGAGTLEHAPHNIVHKWTGLADKPS    253

8BBQ                     228 NNIGGFMQDLLSPTDPVFFAHHSNIDRLWDVWTRKQQRLGLPTLPTGANL    277
                             .::|.|   ..:..||:||.||:|:||:|::|    :.:|      |.|.
2P3X                     254 EDMGNF---YTAGRDPIFFGHHANVDRMWNIW----KTIG------GKNR    290

8BBQ                     278 -----PLWANEPFLFFIGPDGKPVAKNKAGDYATIGDFDYNYE-------    315
                                  ..|.:..|:|:  .:.|.:.|.|..|........|.|:
2P3X                     291 KDFTDTDWLDATFVFY--DENKQLVKVKVSDCVDTSKLRYQYQDIPIPWL    338

8BBQ                     316 PGSGEAV    322
                             |
2P3X                     339 P------    339
```

i) Tyrosinase from *Juglans regia*

```
8BBQ                      1 --------------------------------AKYHRLNLQN------PA     12
                                                            .|...|..||      ||
5CE9                      1 DPVSAPELTLCSEADLPAGALPVNCCPPTSKKIKDFVLPSQNTPLRVRPA     50

8BBQ                     13 A----APFLESYKKAITVMLQLPPSDARNWYRNAFIHTLDCP--------     50
                            |    ..::..|.|.|.:|..||..|.|::.:.|.:|...|.
5CE9                     51 AHLVDNDYIAKYNKGIELMKSLPADDPRSFTQQANVHCAYCDGAYTQVGF    100

8BBQ                     51 -------HGNWWFVVWHRGYTGWFERTVRELSGDPNFAFPYWDWTALP--     91
                                   |..|.|..:||.|..:||:.:.:|.|||.||.|:|:|.:.|
5CE9                    101 PDLSLQIHECWLFFPFHRYYVYFFEKILGKLIGDPTFALPFWNWDSPPGM    150

8BBQ                     92 QVPDSFFNGVLDPNNPAFIASYNEFYSQLSNPMSALWNSFSTAQLQQMRN    141
                            |:|..:                     .:||  ||:::        .:||
5CE9                    151 QLPSLY---------------------AVSN--SAIYD--------PLRN    169

8BBQ                    142 RGFQ--SVNDVWQAVRDSPMFFPRGRARTLTRQNP-GFDATTRRAVSIGT    188
                            ...|  ::.|:        .:.....:.|.|.|.| ......|:.||
5CE9                    170 ANHQPPTIIDL--------DYGETSESTTTTDQVPSNLKIMYRQMVS---    208

8BBQ                    189 IRNALAPTDFITFGSGKTANHSESATQGILESQPHNNVH-----------    227
                              .|..||.|  |||...|........|.:||.||||:|
5CE9                    209 --GAKNPTLF--FGSPYRAGDEPDPGAGTIESTPHNNIHLWTGDDTQPNI    254

8BBQ                    228 NNIGGFMQDLLSPTDPVFFAHHSNIDRLWDVW-TRKQQRLGLPTLPTGAN    276
                            .|:|.|..   :..||:|||||||:||:|.:| |...:|..:..
5CE9                    255 ENMGNFYS---AGRDPIFFAHHSNVDRMWTIWKTLGGKRKDITD------    295

8BBQ                    277 LPLWANEPFLFFIGPDGKPVAKNKAGDYATIGDFDYNYEPGSGEAV----    322
                             |.|.|..| ||...:..|| :.|..|........|.|:.     |
5CE9                    296 -PDWLNSSF-FFYDENADPV-RVKVKDCVDNTKLRYVYQD-----VEIPW    337

8BBQ                    323 --    322

5CE9                    338 LK    339
```

j) Tyrosinase from *Malus domestica*

```
8BBQ                 1 -------------------------------------------AKYHRLN      7
                                                                  ||   |.
6ELS                 1 GPKPIAPPDVSKCGPADLPQGAVPTNCCPPPSTKIIDFKLPAPAK---LR     47

8BBQ                 8 LQNPAAA---PFLESYKKAITVMLQLPPSDARNWYRNAFIHTLDCP----     50
                       ::.||.|   .:.:.|.||:.:|..||..|.|::.:.|.:|...|.
6ELS                48 IRPPAHAVDQAYRDKYYKAMELMKALPDDDPRSFKQQAAVHCAYCDGAYD     97

8BBQ                51 -----------HGNWWFVVWHRGYTGWFERTVRELSGDPNFAFPYWDWTA     89
                                  |.:|.|..:||.|..:||:.:.:|..||.||.|:|:|.:
6ELS                98 QVGFPELELQIHNSWLFFPFHRYYLYFFEKILGKLINDPTFALPFWNWDS    147

8BBQ                90 LPQVPDSFFNGVLDPNNPAFIASYNEFYSQLSNPMSALWNSFSTAQLQ--    137
                       ...:|           .||..|          :|.|.|::...:|..|
6ELS               148 PAGMP-----------LPAIYA----------DPKSPLYDKLRSANHQPP    176

8BBQ               138 QMRNRGFQSVNDVWQAVRDSPMFFPRGRARTLTRQNPGFDATTRRAVSIG    187
                       .:.:..:....|               .....|..|.......|:.||
6ELS               177 TLVDLDYNGTED---------------NVSKETTINANLKIMYRQMVS--    209

8BBQ               188 TIRNALAPTDFITFGSGKTANHSESATQGILESQPHNNVH----------    227
                       ..:||.     :.||:...|........|.:|..||..||
6ELS               210 NSKNAK-----LFFGNPYRAGDEPDPGGGSIEGTPHAPVHLWTGDNTQPN    254

8BBQ               228 -NNIGGFMQDLLSPTDPVFFAHHSNIDRLWDVWTRKQQRLGLPTLPTGAN    276
                        .::|.|..   :..||:|||||||:||:|.:|    :.||      |..
6ELS               255 FEDMGNFYS---AGRDPIFFAHHSNVDRMWSIW----KTLG------GKR    291

8BBQ               277 LPL----WANEPFLFFIGPDGKPVAKNKAGDYATIGDFDYNYEPGSGEAV    322
                       ..|    |.:..|||:  .:...:.:.|..|.....:..|.|:.     |
6ELS               292 TDLTDSDWLDSGFLFY--NENAELVRVKVRDCLETKNLGYVYQD-----V    334

8BBQ               323 --------------------------------------------------    322

6ELS               335 DIPWLSSKPTPRRAKVALSKVAKKLGVAHAAVASSSKVVAGTEFPISLGS    384

8BBQ               323 --------------------------------------------------    322

6ELS               385 KISTVVKRPKQKKRSKKAKEDEEEILVIEGIEFDRDVAVKFDVYVNDVDD    434

8BBQ               323 --------------------------------------------------    322

6ELS               435 LPSGPDKTEFAGSFVSVPHSHKHKKKMNTILRLGLTDLLEEIEAEDDDSV    484

8BBQ               323 ----------------------    322

6ELS               485 VVTLVPKFGAVKIGGIKIEFAS    506
```

k) Tyrosinase from *Agaricus bisporus*

```
8BBQ      1 --------------AKYHRLNL----QNPAAAPFLESYKKAITVMLQLPP     32
                          ...:|||:    :|.   .|...|.:|:.|:.....
2Y9W      1 SDKKSLMPLVGIPGEIKNRLNILDFVKND---KFFTLYVRALQVLQARDQ     47

8BBQ     33 SDARNWYRNAFIHTLD------------------CPHGNWWFVVWHRGYT     64
            ||..::::...||.|.                  |.||...|..|||.|.
2Y9W     48 SDYSSFFQLGGIHGLPYTEWAKAQPQLHLYKANYCTHGTVLFPTWHRAYE     97

8BBQ     65 GWFERTVRELSGDPNFAF-----------------PYWDWTALPQVPDSF     97
            ..:|:|:.|.:|.....|                 |:|||...|..||
2Y9W     98 STWEQTLWEAAGTVAQRFTTSDQAEWIQAAKDLRQPFWDWGYWPNDPD--    145

8BBQ     98 FNGVLDP---NNPAFIASYNEFYSQLSNPMSALWNSFSTAQLQQMRNRGF    144
            |.|:.|.   :....|..||....::.||:  |...|...:      ..|
2Y9W    146 FIGLPDQVIRDKQVEITDYNGTKIEVENPI--LHYKFHPIE------PTF    187

8BBQ    145 QSVNDVWQAVRDSPMFFP--------RGRARTLTRQNPGFDATTRRAVSI    186
            :.....||    :.|.:|        .|....:....|||     |..:.
2Y9W    188 EGDFAQWQ----TTMRYPDVQKQENIEGMIAGIKAAAPGF-----REWTF    228

8BBQ    187 GTIRNALAPTDFITFGS--GKTANHSESATQGILESQPHNNVHNNIGGFM    234
            ..:........|...|:  |..||..|..         ||.||..||  .
2Y9W    229 NMLTKNYTWELFSNHGAVVGAHANSLEMV---------HNTVHFLIG--R    267

8BBQ    235 QDLLSPT-------------DPVFFAHHSNIDRLWDVW------------    259
            ...|.|.             ||:|:.||.|:|||..:|
2Y9W    268 DPTLDPLVPGHMGSVPHAAFDPIFWMHHCNVDRLLALWQTMNYDVYVSEG    317

8BBQ    260 TRKQQRLGL-PTLPTGANLPLWANEPFLFFIGPDGKPVAKNKAGDYATIG    308
            ..::..:|| |.......:.||   ||   |...:..|...:...|:.|:|
2Y9W    318 MNREATMGLIPGQVLTEDSPL---EP---FYTKNQDPWQSDDLEDWETLG    361

8BBQ    309 DFDY-NYEPGSGEAV----------------    322
             |.| :::|..|::.
2Y9W    362 -FSYPDFDPVKGKSKEEKSVYINDWVHKHYG    391
```

I) Pro-tyrosinase from *Aspergillus oryzae*

```
8BBQ                 1 -----------------------------------AKYHRLNLQNPAAAP     15
                                                          |..:|:.|     ..
3W6Q                 1 GPGGSPYLITGIPKDPKHPLPIRKDIDDWYLEQTSAGSNRIQL-----TL     45

8BBQ                16 FLESYKKAITVMLQLPPSDARNWYRNAFIH--------------------     45
                       |:|    |:||:...|.:|..:::|.|.||
3W6Q                46 FVE----ALTVIQNRPLNDQLSYFRLAGIHGAPWTEWDGVPGGQKDSKGN     91

8BBQ                46 -TLDCPHGNWWFVVWHRGYTGWFERTVRELSGD-----------------     77
                        |..|.|.|:.|..|||.|...:|:.:.|...|
3W6Q                92 PTGFCVHNNYTFPTWHRVYVTLYEQVIYEAMLDFIKQNVPQNGKADWENE    141

8BBQ                78 -PNFAFPYWDW---------------------TALPQVPDSFFNGVLDPN    105
                        ..:..||||:                     ..:|.|.      ||.|.
3W6Q               142 AKQWRLPYWDFARFARHGHDNTQGDELRLPILVTMPMVK------VLVPG    185

8BBQ               106 NPA-FIASYNEFYS-QLSNPMSALWNSFS-TAQLQQMRNRGF-------Q    145
                       .|. .::..|..|. |:...|..|...:: |:|..:.....|       |
3W6Q               186 QPGKQLSKPNPLYRFQMQTLMGTLERPYAITSQKTEEHGWSFDLPFDKCQ    235

8BBQ               146 SVN----------DVWQ-----------AVRDSPMFFPRGRARTLTRQN-    173
                       |..          |||.           |:.:.|.:           ||
3W6Q               236 STTKYGLLENYNADVWADGGQNWLRANLALNEHPWY-----------QNL    274

8BBQ               174 PGFDATTRRAVSIGTIRN---ALAPTDFITFGSGKTANHSESATQGILES    220
                       .|:|       |:.|:::   .|..|..:.:|...:.:.:....    |:
3W6Q               275 DGWD-------SVPTLQDMTFRLLTTGGLNWGEFSSTRYDDKKE----ET    313

8BBQ               221 QP----------------HNNVHNNIGGFM------QDL-----------    237
                       ||                ||||||.:||||      .||
3W6Q               314 QPKNNEQAPKNWMNLEAIHNNVHNWVGGFMFSRPGRHDLKLWGAGHMSSV    363

8BBQ               238 -LSPTDPVFFAHHSNIDRLWDVWTRKQQRLGLPTLPTGANLPLWANE---    283
                        ::..||:|:.||.|||||..:|         .|:.:|:    |.|:
3W6Q               364 PVAAYDPIFWLHHCNIDRLTAIW---------QTVNSGS----WFNDDKS    400

8BBQ               284 ---------PF----------LFFIGPDGKPVAKNKAGDYATIGDFDYNY    314
                                ||          :||...|.|        |:.:: ::||
3W6Q               401 KVSKDDDLRPFHRFCEKTRKVVFFRSDDVK--------DWRSL-NYDY--    439

8BBQ               315 EPGSGEAV------------------------------------------    322
                             |:
3W6Q               440 ------AITKDASRIRKEISDLYGQRTKEVYKDFGEEDYILSIRYSRYAL    483

8BBQ               323 --------------------------------------------------    322

3W6Q               484 GGKPFQINIFFGDVDGKDFYDARSQNFVGSVFNFSGSLEDSNCDKCAQQE    533

8BBQ               323 --------------------------------------------------    322

3W6Q               534 QEGVLSVSQLPARLAVHYYKKQNKGEVPTPRYVVVNSQGKAEAEVKVEVA    583

8BBQ               323 -------------------------------------    322

3W6Q               584 LHKTEGTFYDAPARGGSDDYRRVADGKRAEVDDAYRA    620
```

m) Tyrosinase-like protein from *Thermothelomyces thermophila ATCC 42464*

```
8BBQ                1 --------------------------------------------------      0

6Z1S                1 EFRCSSDAPPPAPVGDDLTEPKELTDLFEKAKKAVIDRLHEDEKALRARG     50

8BBQ                1 -------------------AKYHRLNLQNPAAAPFLESYKKAITVMLQLP     31
                                         :|..||            :|..|:..:...|
6Z1S               51 EAPRCTADKLIFRREYGSLSKDERL------------AYVNAVKCLQSKP     88

8BBQ               32 P-------SDARNWYRN-AFIH---TLDCPHGNWWFVVWHRGYTGWFERT     70
                      |       ..||:.:.: ..:|   |||. |.:..|..|||.:...:|:.
6Z1S               89 PRTPASVAPGARSRFDDFVVVHIQQTLDI-HYSGIFQAWHRWFVYQYEKA    137

8BBQ               71 VRELSGDPNFAFPYWDWTALPQVP-DS-FFNGVLDP------------NN    106
                      :|:..|...:. |||||......| || .|||  ||            :.
6Z1S              138 LRDECGYTGYQ-PYWDWPKYASAPQDSPLFNG--DPYSLGGNGEYVPHDG    184

8BBQ              107 PAFIASYNEFYSQLSNPMSALWNSFSTAQLQQMR-NRG-FQSVNDVWQAV    154
                      |..:.........:|.|.........|.....|. |.| ...:.|.....
6Z1S              185 PVIVPPEGVSGGNISLPAGVGGGFVRTGPFANMTVNLGPVGGLADTAPGP    234

8BBQ              155 RDSPMFFPRGRARTLTRQNPGFDATTRRAVSIGTIRNALAPTDFITFGSG    204
                      :....:.|||..|.|     |....||.| :..|:...|...|...|
6Z1S              235 QGGLGYNPRGLKRDL-----GGAMNTRYA-NYTTVLRLLTQPDVDAF---    275

8BBQ              205 KTANHSESATQGI---LESQPHNNVHNNIGGFM-QDLL-SPTDPVFFAHH    249
                            .:.::|:   :|..|||.:|..|||.. .||. ||.||.|:.||
6Z1S              276 ------RTVSEGVPYTVEIGPHNGIHYTIGGDPGGDLFTSPGDPAFWVHH    319

8BBQ              250 SNIDRLWDVWTRKQQRLGLPTLPTGANLPLWANEPFLFFIGPDGKPVAKN    299
                      :.:||:|..|    |.|||  ||...              |.|..|..:
6Z1S              320 AQMDRVWATW----QALGL--LPPAD--------------GGDPDPARR-    348

8BBQ              300 KAGDYATIGDFDYNYEPGSGEAV---------------------------    322
                          |..:|..||.:........
6Z1S              349 ----YTDLGKGDYAHRTWQNSPPSPFAELSDVIDMGYAAPSTTIGAVMST    394

8BBQ              323 -------------------------------    322

6Z1S              395 TEGELCYFYLEQKLISEEDLNSAVDHHHHHH    425
```

n) Tyrosinase from *Agaricus bisporus var. bisporus H97*

```
8BBQ               1 ----------AKYHRLNLQN-PAAAPFLESYKKAITVMLQLPPSDARNWY     39
                               ...:||::.: .....|...|.:|:..:.....||..:::
4OUA               1 XLLATVGPTGGVKNRLDIVDFVRDEKFFTLYIRALQAIQDKDQSDYSSFF     50

8BBQ              40 RNAFIHTLD-------------------CPHGNWWFVVWHRGYTGWFERT     70
                     :.:.||.|.                   |.|....|..|||.|...:|:.
4OUA              51 QLSGIHGLPFTPWAKPKDTPTVPYESGYCTHSQVLFPTWHRVYVSIYEQI    100

8BBQ              71 VRELS----------------GDPNFAFPYWDW----------TALPQVP     94
                     ::|.:                ...:...||||.          ..|.||.
4OUA             101 LQEAAKGIAKKFTVHKKEWAQAAEDLRQPYWDTGFALVPPDEIIKLEQVK    150

8BBQ              95 DSFFNGV-LDPNNPAFIASYNEFYSQLSNPMSALWNSFSTAQLQQMRNRG    143
                     .:.::|. :...||....|:        :|:...:|.:...
4OUA             151 ITNYDGTKITVRNPILRYSF--------HPIDPSFNGYPNF---------    183

8BBQ             144 FQSVNDVWQAVRDSPMFFPRGRARTLTRQNPGFDATTRRAVSIGTIRNAL    193
                          |.|:.                |.:||..|........||.:....
4OUA             184 -----DTWKT----------------TVRNPDADKKENIPALIGKLDLEA    212

8BBQ             194 APTDFITFGSGK-TANHSESATQGILESQPHNN---VHNNIGGFM-----    234
                     ..|...|:...| .||....:..|..:....|:   ||::|.||:
4OUA             213 DSTREKTYNMLKFNANWEAFSNHGEFDDTHANSLEAVHDDIHGFVGRGAI    262

8BBQ             235 -----QDLLSPTDPVFFAHHSNIDR---LWD-----VW-TRKQQRLGLPT    270
                          ..|.:..||:|:.||||:||   ||.     || |:..:|.|...
4OUA             263 RGHMTHALFAAFDPIFWLHHSNVDRHLSLWQALYPGVWVTQGPEREGSMG    312

8BBQ             271 LPTGANLPL-WANEPFLFFIGPDGKPVAKNKAGDYATIG----DFDYNYE    315
                     ...|..|.. .|.||   |...:.||.......|.|.:.    ||| ..:
4OUA             313 FAPGTELNKDSALEP---FYETEDKPWTSVPLTDTALLNYSYPDFD-KVK    358

8BBQ             316 PGSGEAV-----------------    322
                     .|:.:.|
4OUA             359 GGTPDLVRDYINDHIDRRYGIKKS    382
```

o) Tyrosinase-related protein 1 from *Homo sapiens*

```
8BBQ              1 --------------------------------------------------      0

5M8L              1 QFPRQCATVEALRSGMCCPDLSPVSGPGTDRCGSSSGRGRCEAVTADSRP     50

8BBQ              1 --------------------------------------------------      0

5M8L             51 HSPQYPHDGRDDREVWPLRFFNRTCHCNGNFSGHNCGTCRPGWRGAACDQ    100

8BBQ              1 --------------------------------------------------      0

5M8L            101 RVLIVRRNLLDLSKEEKNHFVRALDMAKRTTHPLFVIATRRSEEILGPDG    150

8BBQ              1 --AKYHRLNLQNPAAAPFLESYKKAITVMLQLPPSDARNWYRNAFIHTLD     48
                      .::..:::.|........|.||....:.|           .:| ..:|
5M8L            151 NTPQFENISIYNYFVWTHYYSVKKTFLGVGQ-----------ESF-GEVD    188

8BBQ             49 CPHGNWWFVVWHRGYTGWFERTVRELSGDPNFAFPYWDWTALPQVPDSFF     98
                    ..|....|:.|||.:....|:.::|:..:|:|:.|||::.....|.|...
5M8L            189 FSHEGPAFLTWHRYHLLRLEKDMQEMLQEPSFSLPYWNFATGKNVCDICT    238

8BBQ             99 NGVLDPNNPAFIASYNEFYSQLSNPMSALWNSFSTAQLQQMRNRGFQSVN    148
                    :.::        .|.:.|.|.|.:|.|.    ||..:........:.::.
5M8L            239 DDLM--------GSRSNFDSTLISPNSV----FSQWRVVCDSLEDYDTLG    276

8BBQ            149 DVWQAVRDSPM-FFPRGR-ARTLTRQNPG------------FDATTRRAV    184
                    .:..:..|.|: ..|.|. ||.:.::.|.            ||.....:.
5M8L            277 TLCNSTEDGPIRRNPAGNVARPMVQRLPEPQDVAQCLEVGLFDTPPFYSN    326

8BBQ            185 SIGTIRNALAPTDFITFGSGKTANHSESATQGILESQPHNNVH---NNIG    231
                    |..:.||.:.       |........:.|.:.:     ||..|   |..|
5M8L            327 STNSFRNTVE-------GYSDPTGKYDPAVRSL-----HNLAHLFLNGTG    364

8BBQ            232 GFMQDLLSPTDPVFFAHHSNIDRLWDVWTRKQ----QRLGLPTLPTGAN-    276
                    |  |..|||.||:|...|:..|.::|.|.|:.    ....|...|.|.|
5M8L            365 G--QTHLSPNDPIFVLLHTFTDAVFDEWLRRYNADISTFPLENAPIGHNR    412

8BBQ            277 ----LPLW--ANEPFLFFIGPDGKPVAKNKAGDYATIGDFDYNYE---PG    317
                        :|.|  .....:|...||                :..|.||   |.
5M8L            413 QYNMVPFWPPVTNTEMFVTAPD----------------NLGYTYEIQWPS    446

8BBQ            318 SGEAV    322

5M8L            447 -----    446
```

References

1. Holm, L.; Laiho, A.; Toronen, P.; Salgado, M. DALI shines a light on remote homologs: One hundred discoveries. *Protein Sci* **2023**, *32*, e4519, doi:10.1002/pro.4519.
2. Kampatsikas, I.; Bijelic, A.; Pretzler, M.; Rompel, A. A Peptide-Induced Self-Cleavage Reaction Initiates the Activation of Tyrosinase. *Angew Chem Int Ed Engl* **2019**, *58*, 7475-7479, doi:10.1002/anie.201901332.
3. Molitor, C.; Mauracher, S.G.; Pargan, S.; Mayer, R.L.; Halbwirth, H.; Rompel, A. Latent and active aurone synthase from petals of C. grandiflora: a polyphenol oxidase with unique characteristics. *Planta* **2015**, *242*, 519-537, doi:10.1007/s00425-015-2261-0.
4. Kampatsikas, I.; Bijelic, A.; Rompel, A. Biochemical and structural characterization of tomato polyphenol oxidases provide novel insights into their substrate specificity. *Sci Rep* **2019**, *9*, 4022, doi:10.1038/s41598-019-39687-0.
5. Pluvinage, B.; Grondin, J.M.; Amundsen, C.; Klassen, L.; Moote, P.E.; Xiao, Y.; Thomas, D.; Pudlo, N.A.; Anele, A.; Martens, E.C.; et al. Molecular basis of an agarose metabolic pathway acquired by a human intestinal symbiont. *Nat Commun* **2018**, *9*, 1043, doi:10.1038/s41467-018-03366-x.
6. Hehemann, J.H.; Kelly, A.G.; Pudlo, N.A.; Martens, E.C.; Boraston, A.B. Bacteria of the human gut microbiome catabolize red seaweed glycans with carbohydrate-active enzyme updates from extrinsic microbes. *Proc Natl Acad Sci U S A* **2012**, *109*, 19786-19791, doi:10.1073/pnas.1211002109.
7. Abidi, W.; Zouhir, S.; Caleechurn, M.; Roche, S.; Krasteva, P.V. Architecture and regulation of an enterobacterial cellulose secretion system. *Sci Adv* **2021**, *7*, doi:10.1126/sciadv.abd8049.
8. Ji, S.; Tian, X.; Li, X.; She, Q. Identification and structural analysis of a carbohydrate-binding module specific to alginate, a representative of a new family, CBM96. *J Biol Chem* **2023**, *299*, 102854, doi:10.1016/j.jbc.2022.102854.
9. Morgan, J.L.; McNamara, J.T.; Fischer, M.; Rich, J.; Chen, H.M.; Withers, S.G.; Zimmer, J. Observing cellulose biosynthesis and membrane translocation in crystallo. *Nature* **2016**, *531*, 329-334, doi:10.1038/nature16966.
10. Kuhn, P.; Guan, C.; Cui, T.; Tarentino, A.L.; Plummer, T.H., Jr.; Van Roey, P. Active site and oligosaccharide recognition residues of peptide-N4-(N-acetyl-beta-D-glucosaminyl)asparagine amidase F. *J Biol Chem* **1995**, *270*, 29493-29497, doi:10.1074/jbc.270.49.29493.
11. Jumper, J.; Evans, R.; Pritzel, A.; Green, T.; Figurnov, M.; Ronneberger, O.; Tunyasuvunakool, K.; Bates, R.; Zidek, A.; Potapenko, A.; et al. Highly accurate protein structure prediction with AlphaFold. *Nature* **2021**, *596*, 583-589, doi:10.1038/s41586-021-03819-2.
12. Varadi, M.; Anyango, S.; Deshpande, M.; Nair, S.; Natassia, C.; Yordanova, G.; Yuan, D.; Stroe, O.; Wood, G.; Laydon, A.; et al. AlphaFold Protein Structure Database: massively expanding the structural coverage of protein-sequence space with high-accuracy models. *Nucleic Acids Res* **2022**, *50*, D439-D444, doi:10.1093/nar/gkab1061.
13. Waterhouse, A.M.; Procter, J.B.; Martin, D.M.; Clamp, M.; Barton, G.J. Jalview Version 2--a multiple sequence alignment editor and analysis workbench. *Bioinformatics* **2009**, *25*, 1189-1191, doi:10.1093/bioinformatics/btp033.
14. Madeira, F.; Pearce, M.; Tivey, A.R.N.; Basutkar, P.; Lee, J.; Edbali, O.; Madhusoodanan, N.; Kolesnikov, A.; Lopez, R. Search and sequence analysis tools services from EMBL-EBI in 2022. *Nucleic Acids Res* **2022**, *50*, W276-W279, doi:10.1093/nar/gkac240.
